# Morphological Characteristics of Floral Organs and Their Taxonomic Significance in 23 Species of Bamboo from Southwest China

**DOI:** 10.3390/plants14243751

**Published:** 2025-12-09

**Authors:** Xingyu Wang, Jiaxin Liu, Chongsheng Zhao, Shuguang Wang

**Affiliations:** 1 Biological Research and Utilization Innovation Team in Bamboo Resources of Yunnan Province, Southwest Forestry University, Kunming 650224, China; sevenwangxy@126.com (X.W.);; 2 Science and Technology Innovation Team of National Forestry and Grassland Administration, Southwest Forestry University, Kunming 650224, China; 3 Faculty of Bamboo and Rattan, Southwest Forestry University, Kunming 650031, China

**Keywords:** bamboo, floral organ morphology, taxonomy, principal component analysis, linear discriminant analysis, Southwest China

## Abstract

This study conducted a systematic morphological comparative analysis of reproductive organ structures in 23 bamboo species from Southwest China, focusing on key morphological characteristics including spikelets, florets, lemma, palea, lodicules, pistils, and stamens. Principal component analysis (PCA) and linear discriminant analysis (LDA) were employed for multidimensional variable interpretation. The experimental results demonstrated significant interspecific differences in floral organ morphology among bamboo species; these differences not only aided in species identification but also provided morphological support for clarifying the ambiguous taxonomic boundaries within the *Bambusa–Dendrocalamus–Gigantochloa* (BDG) complex. Spikelet morphology, palea length, and stamen number were identified as core diagnostic indicators for the classification among different bamboo genera. The 11 core traits identified by PCA collectively explained 84.6% of the variation. The LDA further validated the taxonomic reliability of these traits, achieving an overall genus-level classification accuracy of 95.7%. Through quantitative analysis, this research confirmed the critical role of floral morphological characteristics in bamboo classification systems, offering novel morphometric evidence to enhance traditional taxonomic criteria.

## 1. Introduction

Bamboo is a group of plants under the Poaceae family, comprising approximately 120 genera and 1702 species. [1]. It is widely distributed across tropical, subtropical, and temperate regions of all continents except Europe and Antarctica, ranging from lowlands to elevations of approximately 4000 m, playing significant economic and ecological roles in many countries. China serves as a center of bamboo distribution, hosting 34 genera and over 534 species [2].

In fact, the earliest classification of bamboo originated with Rumpf (1750), who categorized bamboo into eight classes, all named under *Arundo*. In 1753, Linnaeus adopted this nomenclature, collectively referring to all bamboos as *Arundo bambos*. Later, the genus name *Bambusa* Schreb. was adopted [3]. In 1788, Retzius first established the sympodial bamboo genus under the name *Bambusa* [4]. In 1803, the first monopodial bamboo genus, *Arundinaria* Michx, was established [5]. Munro (1868) classified global bamboos into three categories based on floral structure and fruit type [6]. Bentham (1881) divided them into four subtribes (Arundinariinae, Sasainae, Dendrocalaminae, and Melocanninae) [7]. Camus (1913) and Camus (1935) expanded the system to five tribes/four subtribes and seven tribes/four subtribes, respectively [8]. Holttum (1956) proposed four functional types based on ovary morphology: Bambusa-type, Oxyten stamena-type, Dendrocalamus-type, and Arundinaria-type [3]. Geng (1957) pioneered a system centered on spikelets (Arundinaria series) and true inflorescences (Bambusa series), with further subdivisions based on spikelet characteristics [9]. Subsequently, Geng revised this into two supertribes and refined tribal classifications [10], while Clayton and Renvoize (1986) proposed a three-subtribe system based on inflorescence type and ovarian appendages [11]. Since the 21st century, molecular phylogenetics had revolutionized bamboo classification, resolving long-standing controversies over generic delimitation and evolutionary relationships. Li suggested that RAPD clustering aligned more closely with traditional classification, according to the cluster analysis of genetic distance among 32 bamboo species in the Sasainae subtribe using RAPD, SSR, and ISSR markers [8].

In plant taxonomy, despite advances in molecular techniques, morphological characteristics remained indispensable for species identification. Peng revealed through nuclear DNA sequence analysis that most genera of temperate bamboos were non-monophyletic, morphological subdivisions at the subtribal level lack support, and molecular results for *Neomicrocalamus prainii* (Gamble) Keng f. exhibited significant inconsistencies with morphologically similar temperate taxa [12]. Zhang et al. reconstructed the molecular phylogeny of 146 representative species in the Arundinarieae tribe using eight chloroplast DNA fragments, finding substantial discrepancies with classical classifications [13]. Subsequently, by sampling 108 species from 25 genera within this tribe and utilizing single-copy nuclear gene GBSSI sequences, their research demonstrated conflicts between gene trees and morphological classifications, necessitating a re-evaluation of the taxonomic significance of certain key morphological traits [13]. These findings indicated that molecular classification methods for bamboos did not fully align with morphological classification results. However, the prolonged and unpredictable flowering cycles of bamboos (from decades to a century) result in scarce floral specimens, forcing traditional classifications to rely heavily on vegetative characteristics, which were subject to considerable debate [14]. For instance, *Dendrocalamus giganteus* Wall. ex Munro was once classified under *Bambusa*, but molecular evidence later confirmed its genetic independence [15,16,17].

In the classification research of bamboo plants, the taxonomic status of *Neosinocalamus* Keng f. and *Dendrocalamopsis* (Chia et Fang) Keng f. had long been subjected to numerous controversies and difficulties. McClure established the genera *Lingnania* and *Sinocalamus* in 1940, and Chia adjusted this classification, assigning some species of *Sinocalamus* to *Dendrocalamus* and placing the remaining species in *Bambusa* in 1980. However, Keng reorganized them, establishing the genera *Dendrocalamopsis* and *Neosinocalamus* in 1983 [18]. This process clearly illustrated the complexity and variability of classification, jointly revealing the difficulty and intricacy of taxonomic work, as well as their shifting classifications between *Dendrocalamus* and *Bambusa*. Both *Flora Reipublicae Popularis Sinicae* [19] and *Flora of China* [2] also documented in detail the complex changes in the taxonomic status of *Neosinocalamus* and *Dendrocalamopsis*. For example, *Bambusa emeiensis* L. C. Chia & H. L. Fung (formerly *Neosinocalamus affinis* (Rendle) P. C. Keng) was once classified under *Dendrocalamus*, and *Dendrocalamopsis* had also undergone taxonomic changes, such as being classified as a subgenus under *Bambusa* [19].

Since the 20th century, molecular systematics techniques have provided new tools for resolving such controversies. Kelchner and Clark, through chloroplast *rpl16* intron sequence analysis, revealed evolutionary branching within the Bambusoideae subfamily, confirming the critical role of floral characteristics in generic differentiation [20]. Vorontsova et al. integrated morphological and molecular data to divide the Bambusoideae into 3 tribes and 18 subtribes, offering a framework reference for global bamboo classification [21]. Nevertheless, molecular approaches had also exposed widespread genetic introgression and polyphyletic or paraphyletic patterns in bamboo taxonomy, as well as resolution difficulties caused by convergent gene sequences. These issues were particularly pronounced in the *Bambusa–Dendrocalamus–Gigantochloa* (BDG) complex [12].

In recent years, with increasing reports of flowering bamboo species in China, research on floral morphology structures has expanded, focusing on floral organ development and gametogenesis in species such as *Phyllostachys violascens* (Carrière) Riviere & C. Rivière [22], *Dendrocalamus sinicus* L. C. Chia & J. L. Sun [23], *Shibataea chinensis* Nakai [24], *Bambusa multiplex* (Lour.) Raeusch. ex Schult. & Schult. f. [25], *Bambusa eutuldoides* var. *viridivittata* (W. T. Lin) L. C. Chia [26], *Bambusa intermedia* Hsueh & T.P. Yi [27], *Neomicrocalamus prainii* (Gamble) Keng f [28], *Bambusa oldhamii* Munro [29], and *Bambusa rigida* Keng & Keng f [30]. However, systematic comparisons of floral traits across genera remained scarce. Du et al. [31] systematically documented the flowering and fruiting patterns of 61 bamboo species in Yunnan province, providing a valuable foundation for regional research. Nevertheless, existing studies predominantly focused on single or few bamboo species, leaving a scarcity of systematic comparative research on floral characteristics across genera. Floral morphology for some significant bamboo species even remained undocumented in the literature [19]. Furthermore, floral traits, owing to their evolutionary conservatism, had been demonstrated to align closely with molecular phylogenies, holding unique value in resolving taxonomic disputes and identifying hybrids [32].

This study selected 23 representative bamboo species from Southwest China, focusing on seven genera: *Bambusa*, *Dendrocalamus*, *Fargesia*, *Gigantochloa*, *Phyllostachys*, *Pleioblastus*, and *Schizostachyum*. The *Bambusa–Dendrocalamus–Gigantochloa* (BDG) complex within this group is taxonomically contentious due to morphological overlap and conflicting molecular signals. The aim of this study was to systematically compare floral morphological characteristics across species, thereby addressing data gaps for under-described species like *Pleioblastus fortunei*, *Gigantochloa* sp. 1, *Gigantochloa* sp. 2, *Phyllostachys sulphurea*, and *Fargesia yuanjiangensis*. Multidimensional floral trait data were integrated and analyzed using principal component analysis (PCA) to identify key diagnostic indicators. A linear discriminant analysis (LDA) model was then constructed to provide morphological support for genus-level classification, especially within the BDG complex. Through integrated morphological and numerical analyses, this research investigates the structural characteristics of bamboo floral organs and explores the use of multivariate statistical methods to provide quantitative morphological criteria for bamboo taxonomy.

## 2. Results

**Figure 1:**
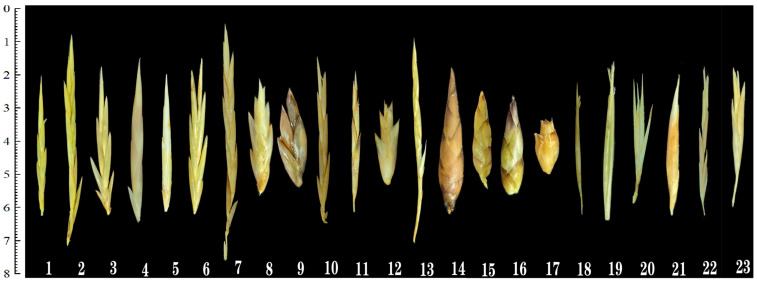
**Morphology of the spikelet of different bamboo species.** 1. *Bambusa sinospinosa.* 2. *Bambusa ventricosa.* 3. *Bambusa eutuldoides var. viridivittata.* 4. *Bambusa tuldoides.* 5. *Bambusa textilis.* 6. *Bambusa rigida.* 7. *Bambusa rutila.* 8. *Bambusa emeiensis.* 9. *Bambusa cerosissima.* 10. *Bambusa intermedia.* 11. *Gigantochloa* sp. 12. *Gigantochloa* sp. 13. *Pleioblastus fortunei.* 14. *Dendrocalamus sinicus.* 15. *Dendrocalamus giganteus.* 16. *Dendrocalamus fugongensis.* 17. *Dendrocalamus hamiltonii.* 18. *Phyllostachys sulphurea.* 19. *Phyllostachys glauca.* 20. *Phyllostachys nigra.* 21. *Schizostachyum brachycladum.* 22. *Fargesia yuanjiangensis.* 23. *Fargesia fungosa*.

Bamboo flowers, a unique morphological stage in the bamboo life cycle, hold significant value for species classification and botanical research. The spikelet serves as a fundamental composite unit of bamboo inflorescence, comprising multiple florets and glumes(Figure 1). The spikelet of bamboo consists of a rachilla (a branch of the inflorescence axis) and florets borne on the axis. The rachilla belongs to the inflorescence system, while the florets include floral organs such as the palea, lemma, stamens, pistil, and lodicules. Notably, these protective structures exhibit interspecific variation in shape among different bamboo species. Furthermore, quantitative and morphological differences in reproductive organs (androecium, lodicules, and gynoecium) serve as critical taxonomic identifiers across bamboo varieties.

### 2.1. Morphological Structure and Characteristics of Bamboo Flowers

From 2010 to 2024, detailed observations and records were conducted on the flowering phenomena of 23 bamboo species in Yunnan, Guizhou, and other regions of China. The floral organs were morphologically examined and their characteristic features documented (Table 1), including ten species from *Bambusa*, two from *Gigantochloa*, one from *Pleioblastus*, four from *Dendrocalamus*, three from *Phyllostachys*, one from *Schizostachyum*, and two from *Fargesia*. Among the 23 bamboo species whose flowers were dissected, a small number exhibited morphological differences from the descriptions provided in *Flora of China* and other literature. Additionally, detailed floral morphological records for several species were not available in previous studies. Notably, two *Gigantochloa* specimens, representing new varieties awaiting formal publication, were included in this analysis. These specimens, temporarily designated as *Gigantochloa* sp. 1 and *Gigantochloa* sp. 2, provide the first comparative floral morphological data for the genus in this region. They have been deposited in the Herbarium of Southwest Forestry University (SWFC), Kunming, China, under voucher numbers SWFC0072425 and SWFC0072426, respectively, and are publicly accessible for verification and future reference. All collected bamboo species were artificially cultivated, with most specimens rarely producing seeds during the collection period.

**Figure 2:**
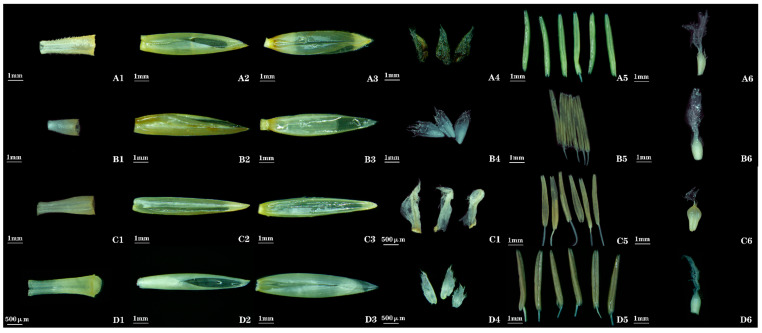
**Morphology of floral organs in *Bambusa* bamboos. A1**, **B1**, **C1**, **D1**: rachilla. **A2**, **B2**, **C2**, **D2**: lemma. **A3**, **B3**, **C3**, **D3**: palea. **A4**, **B4**, **C4**, **D4**: lodicules. **A5**, **B5**, **C5**, **D5**: stamens. **A6**, **B6**, **C6**, **D6**: gynoecium. **A1**–**A6**: B. sinospinosa. **B1**–**B6**: B. ventricosa. **C1**–**C6**: B. eutuldoides. **D1**–**D6**: B. tuldoides.

***Bambusa sinospinosa* McClure**: The floral morphology is basically consistent with the description in *Flora of China*, but there are differences in the length of the lemma/palea, the shape of the lemma, and the shape of the ovary compared with the records in *Flora of China* (see Table 1 for complete data). In this study, the lemma length was measured to be 7.3–8.2 mm, and the palea length was 7.1–9.0 mm; the palea is slightly longer than or equal in length to the lemma, the lemma is ovate-lanceolate (Figure 2 (A2)), and the ovary is obovate (Figure 2 (A6)). However, *Flora of China* records that the lemma is longer than the palea, the lemma is ovate-oblong, and the ovary is narrowly elongated.

***Bambusa ventricosa* McClure**: Its floral morphology is basically consistent with the records in *Flora of China*, but there are differences in the lengths of the lemma/palea, the shape of the lemma, and the shape of ovary compared with the descriptions in *Flora of China* (see Table 1 for complete data). In this study, the measured lemma length ranges from 9.2–10.5 mm, and the palea length ranges from 9.7–11.3 mm; the palea is slightly longer than the lemma, the lemma is ovate-lanceolate (Figure 2 (B2)), and the ovary is obovate (Figure 2 (B6)). However, *Flora of China* records that the lemma is equal in length to the palea, the lemma is ovate-elliptic, and the ovary is narrowly elongated.

***Bambusa eutuldoides* var. *viridivittata* (W. T. Lin) L. C. Chia**: Its floral morphology is basically consistent with the literature records, but there are differences in the lengths of the lemma/palea, the shape of the lodicules, and the shape of the ovary compared with the descriptions by Tang et al. [5] (see Table 2 for complete data). In this study, the measured lemma length ranges from 9.4–11.0 mm, and the palea length ranges from 9.7–11.1 mm; the palea is slightly longer than the lemma, the lodicules are ovate-lanceolate (Figure 2 (C4)), and the ovary is obovate (Figure 2 (C6)). However, Tang et al. [5] described that the lemma is equal in length to the palea, the lodicules are obovate, and the ovary is ovoid.

***Bambusa tuldoides* Munro**: Its floral morphology is basically consistent with the records in *Flora of China*, but there is a difference in the shape of the lemma compared with the description in *Flora of China* (see Table 1 for complete data). In this study, the lemma was observed to be ovate-lanceolate (Figure 2 (D2)); however, *Flora of China* records that the lemma is ovate-oblong.

**Figure 3:**
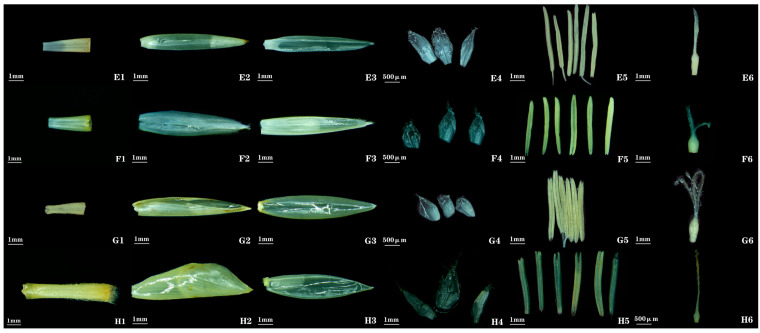
**Morphology of floral organs in *Bambusa* bamboos. E1**, **F1**, **G1**, **H1**: rachilla. **E2**, **F2**, **G2**, **H2**: lemma. **E3**, **F3**, **G3**, **H3**: palea. **E4**, **F4**, **G4**, **H4**: lodicules. **E5**, **F5**, **G5**, **H5**: stamens. **E6**, **F6**, **G6**, **H6**: gynoecium. **E1**–**E6**: *B. textilis*. **F1**–**F6**: *B. rigida*. **G1**–**G6**: *B. rutila*. **H1**–**H6**: *B. emeiensis*.

***Bambusa textilis* McClure**: Its floral morphology is basically consistent with the records in *Flora of China*, but there are differences in the lengths of the lemma/palea, the shape of the lemma, and the shape of the ovary compared with the descriptions in *Flora of China* (see Table 1 for complete data). In this study, the measured lemma length ranges from 12.3–14.5 mm, and the palea length ranges from 12.3–13.7 mm; the palea is slightly shorter than or equal in length to the lemma, the lemma is ovate-lanceolate (Figure 3 (E2)), and the ovary is obovate (Figure 3 (E6)). However, *Flora of China* records that the lemma is slightly shorter than the palea, the lemma is elliptic, and the ovary is broadly ovoid.

***Bambusa rigida* Keng & P. C. Keng**: Its floral morphology is basically consistent with the records in *Flora of China*, but there is a difference in the shape of the ovary compared with the description in *Flora of China* (see Table 1 for complete data). In this study, the ovary was observed to be obovate (Figure 3 (F6)); however, *Flora of China* records that the ovary is ovoid.

***Bambusa rutila* McClure**: Floral morphology of *Bambusa rutila* agrees with *Flora of China*; no additional traits were observed (Figure 3 (G)).

***Bambusa emeiensis* L. C. Chia & H. L. Fung**: Morphological features such as the spikelet shape, and ovary shape of this bamboo species have been described less frequently in previous studies. Spikelets are long-ovate (Figure 1 (8)). Ovary is broadly obovate, sparsely pubescent; stigmas 3, plumose, exhibiting a long-style/long-stigma type (Figure 3 (H6)).

**Figure 4:**
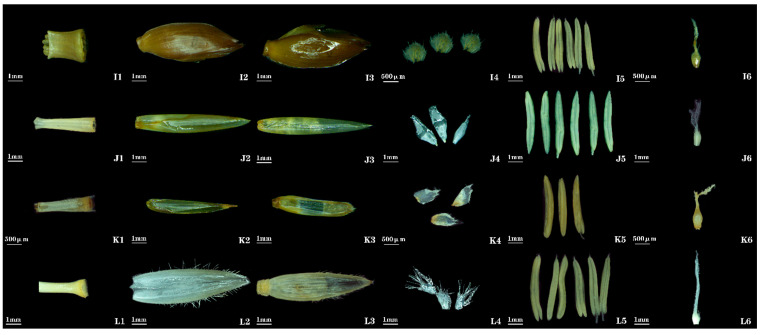
**Floral organ morphology of *Bambusa* and *Gigantochloa* bamboos. I1**, **J1**, **K1**, **L1**: rachilla. **I2**, **J2**, **K2**, **L2**: lemma. **I3**, **J3**, **K3**, **L3**: palea. **I4**, **J4**, **K4**, **L4**: lodicules. **I5**, **J5**, **K5**, **L5**: stamens. **I6**, **J6**, **K6**, **L6**: gynoecium. **I1**–**I6**: *B. cerosissima*. **J1**–**J6**: *B. intermedia*. **K1**–**K6**: *Gigantochloa* sp. **L1**–**L6**: *Gigantochloa* sp.

***Bambusa cerosissima* McClure**: Floral morphology of *Bambusa cerosissima* agrees with *Flora of China*; no additional traits were observed (Figure 4 (I)).

***Bambusa intermedia* J. R. Xue & T. P. Yi**: Morphological features such as spikelet shape, lemma shape, ovary shape, and lodicule characteristics of this bamboo species have been described less frequently in previous studies. Spikelets are linear-lanceolate, (Figure 1 (10)), and the Lemma is ovate-oblong (Figure 4 (J2)). Lodicules 3, slender-lanceolate (Figure 4 (J4)). Ovary is obovoid, pubescent on the upper part; stigmas 3, plumose, exhibiting a short-style/short-stigma type (Figure 4 (J6)).

***Gigantochloa* sp. 1**: This bamboo species has not been formally published. Spikelets are oblong-lanceolate, slightly flattened, 2.83 cm long, 2.1 mm wide, and clustered at each node of flowering branches. Bud bracts 2–3, narrowly ovate, apex abruptly acute. Spikelets contain 5–7 florets (Figure 1 (11)). Rachilla is nearly solid (Figure 4 (K1)). Lemma is oblong-lanceolate, 7.58 mm long, glabrous, and apex abruptly acute (Figure 4 (K2)). Palea is linear, slightly shorter than lemma (5.75 mm long), bearing short cilia along keel, and apex truncate with ciliate margins (Figure 4 (K3)). Lodicules 3, ovate-lanceolate (Figure 4 (K4)). Stamens 3; filaments slender; stamens tipped with white brush-like hairs (Figure 4 (K5)). Ovary is obovate; stigmas 3, plumose, exhibiting a short-style/long-stigma type (Figure 4 (K6)).

***Gigantochloa* sp.** 2: This bamboo species has not yet been formally published. Spikelets are oblong, slightly flattened, 2.25 cm long, 6.11 mm wide, and densely clustered or forming spherical clusters at nodes of flowering branches. Bud bracts 1–2, ovate, apex blunt with a mucronate tip. Spikelets contain 4–5 florets (Figure 1 (12)). Rachilla is nearly solid (Figure 4 (L1)). Lemma is broadly ovate, 9.39 mm long, margins ciliate, and apex abruptly acute (Figure 4 (L2)). Palea is slightly shorter than lemma, 7.82 mm long, with short cilia along keels (Figure 4 (L3)). Lodicules 3, ovate-lanceolate (Figure 4 (L4)). Stamens 6; filaments slender (Figure 4 (L5)). Ovary is obovate, apex thickened, and covered with short stiff hairs; stigmas 3, feathery, exhibiting a short-style/long-stigma type (Figure 4 (L6)).

**Figure 5:**
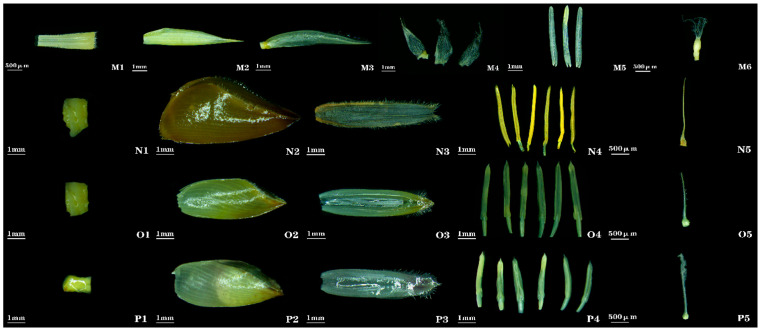
**Floral organ morphology of *Pleioblastus* and *Dendrocalamus* bamboos**. **M1**, **N1**, **O1**, **P1**: rachilla. **M2**, **N2**, **O2**, **P2**: lemma. **M3**, **N3**, **O3**, **P3**: palea. **M4**: lodicules. **M5**, **N4**, **O4**, **P4**: stamens. **M6**, **N5**, **O5**, **P5**: gynoecium. **M1**–**M6**: *P. pygmaeus*. **N1**–**N5**: *D. sinicus*. **O1**–**O5**: *D. giganteus*. **P1**–**P5**: *D. fugongensis*.

***Pleioblastus fortunei* (Van Houtte ex Munro) Nakai**: Spikelets are linear-lanceolate, slightly flattened, 7.03 cm long, 2.44 mm wide, and clustered at nodes of flowering branches. Bud bracts 1–2, narrowly ovate, apex sharply pointed. Spikelets contain 4–9 florets (Figure 1 (13)). Rachilla is nearly solid (Figure 5 (M1)). Lemma is lanceolate, 13.63 mm long, and apex sharply pointed (Figure 5 (M2)). Palea is slightly shorter than lemma (12.63 mm long), with short cilia near apical margins (Figure 5 (M3)). Lodicules 3, oblong-lanceolate, and margins ciliate (Figure 5 (M4)). Stamens 3; filaments slender (Figure 5 (M5)). Ovary iselliptical; style short; stigmas 3, feathery, exhibiting a short-style/long-stigma type (Figure 5 (M6)). Floral characteristics of this species were not documented in *Flora of China*.

***Dendrocalamus sinicus* L. C. Chia & J. L. Sun**: Previous studies have provided limited descriptions concerning the spikelet morphology, lemma shape, and ovary shape of this bamboo species. Spikelets are ovoid with tapering apices (Figure 1 (14)). Rachilla is nearly solid (Figure 5 (N1)). Lemma is broadly ovate (Figure 5 (N2)). Ovary is globular, with apical pubescence extending to the stigma; stigma is solitary, feathery, exhibiting a long-style/long-stigma type (Figure 5 (N5)).

***Dendrocalamus giganteus* Wall. ex Munro**: Floral morphology of *Dendrocalamus giganteus* agrees with *Flora of China*; no additional traits were observed (Figure 5 (O)).

***Dendrocalamus fugongensis* J. R. Xue & D. Z. Li**: Floral morphology of *Dendrocalamus fugongensis* agrees with *Flora of China*; no additional traits were observed (Figure 5 (P)).

**Figure 6:**
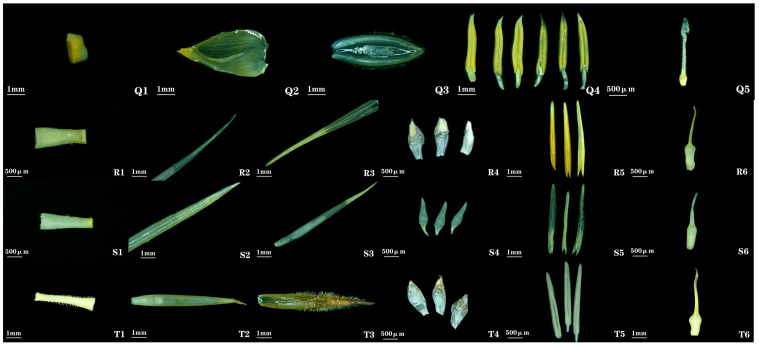
**Floral organ morphology of *Dendrocalamus* and *Phyllostachys* bamboos**. **Q1**, **R1**, **S1**, **T1**: rachilla. **Q2**, **R2**, **S2**, **T2**: lemma. **Q3**, **R3**, **S3**, **T3**: palea. **R4**, **S4**, **T4**: lodicules. **Q4**, **R5**, **S5**, **T5**: stamens. **Q5**, **R6**, **S6**, **T6**: gynoecium. **Q1**–**Q5**: *D. hamiltonii*. **R1**–**R6**: *P. sulphurea*. **S1**–**S6**: *P. glauca*. **T1**–**T6**: *P nigra*.

***Dendrocalamus hamiltonii* Nees & Arn. ex Munro**: The spikelet shape, lemma shape, and ovary morphology of this bamboo species lacked detailed descriptions in previous research reports. Spikelets are ovate with obtuse apex, slightly flattened (Figure 1 (17)). Rachis is nearly solid (Figure 6 (Q1)). Lemma is broadly ovate (Figure 6 (Q2)). Ovary is globular; style is exceptionally long; stigma is solitary, feathery, exhibiting a long-style/long-stigma type (Figure 6 (Q5)).

***Phyllostachys sulphurea* (Carrière) Riviere & C. Rivière**: Floral characteristics were not documented in *Flora of China*. This species was commonly found in southwestern China. Spikelets are narrowly lanceolate, 2.75 cm long, 1.50 mm wide, borne at the apex of branches, and enclosed by a spathe formed from a single leaf sheath. Bud bracts 3–5, lanceolate, with pubescent apices. Spikelets contain 3–5 florets (Figure 1 (18)). Rachis is nearly solid (Figure 6 (R1)). Lemma is lanceolate, 19.25 mm long, apex sharply pointed, sparsely pubescent abaxially, glabrous adaxially (Figure 6 (R2)). Palea is nearly equal to or slightly longer than lemma, 20.51 mm long, apex acute, and densely pubescent along keels (Figure 6 (R3)). Lodicules 3, subequal, lanceolate, ciliate at apex (Figure 6 (R4)). Stamens 3; filaments slender (Figure 6 (R5)). Ovary is obovate; stigma solitary, sparsely plumose, exhibiting a long-style/short-stigma type (Figure 6 (R6)).

***Phyllostachys glauca* McClure**: The shapes of the lemma, ovary, and lodicules of this bamboo species lacked detailed descriptions in previous studies. Lemma is lanceolate, (Figure 6 (S2)). Lodicules 3, lanceolate, ciliate at apex (Figure 6 (S4)). Ovary is obovate; stigma solitary, plumose, exhibiting a long-style/short-stigma type (Figure 6 (S6)).

***Phyllostachys nigra* (Lodd. ex Lindl.) Munro**: The shape of the lemma, ovary, and lodicules in this bamboo species lacked detailed descriptions in previous studies. Lemma is lanceolate, (Figure 6 (T2)). Lodicules 3, subequal, obovate, ciliate at apex (Figure 6 (T4)). Ovary is obovate; stigma solitary, plumose, exhibiting a long-style/short-stigma type (Figure 6 (T6)).

**Figure 7:**
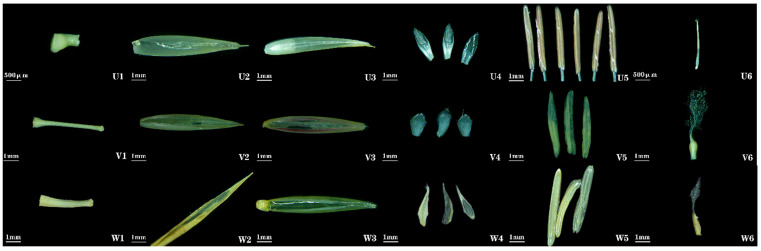
**Floral organ morphology of *Schizostachyum* and *Fargesia* bamboos. U1**, **V1**, **W1**: rachilla. **U2**, **V2**, **W2**: lemma. **U3**, **V3**, **W3**: palea. **U4**, **V4**, **W4**: lodicules. **U5**, **V5**, **W5**: stamens. **U6**, **V6**, **W6**: gynoecium. **U1**–**U6**: *S. brachycladum*. **V1**–**V6**: *F. yuanjiangensis*. **W1**–**W6**: *F. fungosa*.

***Schizostachyum brachycladum* (Kurz ex Munro) Kurz**: Floral characteristics were not documented in *Flora of China*. Spikelets are lanceolate, 2.75 cm long, 3.32 mm wide, clustered at nodes on flowering branches. Bud bracts 1–2, broadly ovate, apex shortly pointed. Spikelets contain 2–3 florets (Figure 1 (21)). Rachis is nearly solid (Figure 7 (U1)). Lemma is ovoid-caudate, 11.33 mm long, apex abruptly mucronate, glabrous externally (Figure 7 (U2)). Palea is slightly shorter than lemma, 7.56 mm long, apex similarly acute, nearly glabrous along keel (Figure 7 (U3)). Lodicules 3, subequal, oblong-lanceolate, ciliate at apex (Figure 7 (U4)). Stamens 6; filaments slender (Figure 7 (U5)). Ovary is broadly ovate; stigma solitary, sparsely feathery, exhibiting a long-style/short-stigma type (Figure 7 (U6)).

***Fargesia yuanjiangensis* J. R. Xue & T. P. Yi**: Floral characteristics were not documented in *Flora of China*. Spikelets are linear-lanceolate, 3.98 cm long, 2.52 mm wide, clustered at nodes on flowering branches. Bud bracts 2, lanceolate, apex acute. Spikelets contain 4–6 florets (Figure 1 (22)). Rachilla are nearly solid (Figure 7 (V1)). Lemma is lanceolate, 9.42 mm long, apex gradually acuminate, margins ciliate (Figure 7 (V2)). Palea is nearly equal to lemma, 8.67 mm long, keels ciliate, apex truncate (Figure 7 (V3)). Lodicules 3, ovate, apical margins ciliate (Figure 7 (V4)). Stamens 3 (Figure 7 (V5)). Ovary is nearly obovate; stigmas 3, feathery, exhibiting a short-style/long-stigma type (Figure 7 (V6)).

***Fargesia fungosa* T. P. Yi**: Floral morphology of *Fargesia fungosa* agrees with *Flora of China*; no additional traits were observed (Figure 7 (W)).

Through morphology observations of the floral organs from 23 bamboo species across 7 genera in southwestern China, significant morphological differences were identified among genera. The *Bambusa* genus exhibited linear-lanceolate spikelets with three distinct lodicules (obovate or lanceolate), six stamens, nearly hollow rachises, and predominantly short-styled pistils with long stigmas (e.g., *Bambusa sinospinosa, Bambusa pervariabilis*). The *Dendrocalamus* genus was characterized by ovate spikelets, complete absence of lodicules, six stamens, nearly solid rachises, and long-styled pistils with elongated stigmas. *Phyllostachys* displayed narrow-lanceolate spikelets enclosed by spathes, three lanceolate lodicules, three stamens, nearly solid rachises, and long-styled pistils with short stigmas. *Fargesia* species showed linear-lanceolate spikelets with three ovate lodicules, three stamens, nearly solid rachises, and short-styled pistils with long stigmas. The *Gigantochloa* genus exhibited significant interspecific variation in stamen number (3 or 6), lanceolate spikelets, three lodicules, and nearly solid rachises. Among these bamboos, the *Bambusa* species represented the most extensively collected specimens, followed by *Dendrocalamus* and *Phyllostachys*, which correlated with the quantitative distribution of bamboo species in the southwestern region. Notably, the floral morphological characteristics of species including *Pleioblastus fortunei*, *Gigantochloa* sp., *Gigantochloa* sp., *Phyllostachys sulphurea*, and *Fargesia yuanjiangensis* were not documented in the *Flora of China*, *Illustrated Monograph of Chinese Bamboos*, or other existing literature. This study provided critical evidence for future taxonomic identification and classification of bamboos.

### 2.2. Principal Component Analysis of Phenotypic Traits in Bamboo Flowers

Based on the observation and analysis of bamboo flower morphological structures, 16 floral organ phenotypic traits (eight qualitative traits and eight quantitative traits) were selected for principal component analysis. Using an eigenvalue λ > 1 as the extraction threshold, five principal components were extracted, primarily composed of 11 correlated floral traits, with a cumulative variance contribution rate of 84.6%, reflecting the majority of information from the original floral dataset (Table 2).

The first principal component (PC1) explained 35.4% of the variance. PC1 integrated traits primarily reflected spikelet shape and lodicule number. PC2 explained 23.5% of the variance and was significantly influenced by spikelet width, stamen number, and lodicule shape. PC3 contributed 11.2% of the variance mainly through spikelet length, lemma length, palea length, and the presence or absence of pubescence on the palea margin. PC4 contributed 8.1% of the variance and partially influenced the presence or absence of pubescence on the lemma margin and palea margin. PC5 primarily explained 6.4% of the variance in spikelet length, spikelet width, and ovary shape. In summary, spikelet morphology, palea length, and stamen number were the most significant traits influencing the morphological variation in bamboo flowers.

### 2.3. Linear Discriminant Analysis of Bamboo Floral Phenotypic Traits

Based on the principal component analysis (PCA) results (Table 2), 11 core traits with absolute loadings ≥ 0.35 were selected: spikelet morphology, spikelet length, spikelet width, lemma length, lemma margin pubescence presence, palea length, palea margin pubescence presence, ovary shape, stamen number, lodicule number, and lodicule shape. Using these 11 high-loading traits as input variables and “genus” as the grouping factor, a linear discriminant analysis (LDA) classification model was constructed. Model efficacy was evaluated via original classification accuracy and leave-one-out cross-validation accuracy. LDA extracted six discriminant functions, with the first two cumulatively explaining 89.7% of the total variance, indicating strong discriminative power (Table 3). Results demonstrated that the 11 high-loading traits significantly distinguished bamboo genera. Among these, spikelet morphology, palea length, and stamen number were core traits distinguishing generic-level taxa (Table 4). The cross-validation accuracy reached 95.7% (Table 5), confirming that the LDA model based on PCA high-loading traits effectively classifies bamboo at the genus level, providing morphological evidence for bamboo systematic taxonomy.

## 3. Discussion

The evolutionary trajectory of the classification system for Bambusoideae reveals that early taxonomic practices predominantly relied on vegetative organ characteristics, such as rhizome types and branching patterns. Modern molecular phylogenetic studies, however, have confirmed the irreplaceable role of floral characteristics in elucidating intergeneric relationships [32]. The florets of bamboo typically comprise five components: lemma, palea, lodicules, stamens, and pistil, which exhibit interspecific variations in morphology, number, and coloration [33]. Through morphological investigations of floral organs across 23 bamboo species in southwestern China, this study unveils the diversity and complexity of floral structures among different species.

Prior research has documented the floral morphology of species such as *Dendrocalamus sinicus* [23], *Bambusa eutuldoides* [24], *Bambusa intermedia* [27], and *Bambusa rigida* [30], with observations in this study aligning consistently with earlier descriptions. Comparative analyses demonstrated significant morphological and structural divergence in floral organs across species. These distinctions not only facilitate species identification but also provide critical insights for systematic taxonomy, enabling comprehensive descriptions of bamboo classification and offering an intuitive understanding of floral morphology across diverse species.

Through systematic morphological observations of floral organs in 23 bamboo species from Southwest China, combined with multivariate statistical methods including principal component analysis (PCA) and linear discriminant analysis (LDA), the research revealed that key traits identified by PCA—such as spikelet morphology, palea length, and stamen number—collectively explained 84.6% of the observed variations. LDA further validated the taxonomic reliability of these traits, achieving an overall genus-level classification accuracy of 95.7%. Specifically, the discrimination accuracy reached 100% for *Bambusa* (10 species) and 75% for *Dendrocalamus* (4 species). These results not only confirm the stability of floral characteristics as critical markers for defining bamboo genera but also provide practical quantitative indicators for rapid field identification. This offers new morphological support for resolving long-debated taxonomic boundaries within the BDG complex group. The findings demonstrated that comparative morphological analysis of floral organs across genera, integrated with multivariate statistical methods like PCA and LDA, effectively addressed the limitations of traditional bamboo classification reliant on vegetative traits. Nevertheless, it must be emphasized that the long and highly unpredictable flowering cycles of bamboos severely restrict the routine application of floral characters in everyday identification. During our 14-year sampling period in south-west China, only a small fraction of bamboo taxa produced inflorescences; the vast majority still lack any floral material. Consequently, this study does not advocate replacing traditional vegetative keys with reproductive traits; rather, we offer a quantitative, repeatable ancillary tool that can be employed whenever flowering material becomes available. Future taxonomic work should continue to rely primarily on vegetative characteristics, while integrating floral data and molecular evidence for critical or problematic taxa in order to enhance accuracy and stability.

*D. giganteus* was previously misclassified into the genus *Bambusa* due to similarities in culm morphology and branching characteristics [2]. However, this study unequivocally demonstrated through the analysis of LDA that significant, non-negligible differences existed between them in lodicule characteristics (*D. giganteus* lacked lodicules, while *Bambusa* possessed three obovate lodicules) and spikelet length-to-width ratio (approximately 0.5 for *D. giganteus* vs. about 10 for *Bambusa*). These morphological evidences corroborated the molecular phylogenetic findings of Kelchner et al. [20], providing further support for the taxonomic placement of *D. giganteus* within *Dendrocalamus*.

Particular attention was warranted regarding the taxonomic placement of *B. emeiensis*. Significant divergence had long persisted concerning its systematic position, with the core controversy centering on the uniqueness of its vegetative organ characteristics and its relationship to closely related taxa. Scholars represented by Bojie Geng argued that *B. emeiensis* exhibited distinct differences from typical sympodial caespitose *Bambusa* species and was insufficiently similar to justify its inclusion within that genus, primarily based on vegetative traits such as its sympodial leptomorph rhizomes (with well-developed pseudorhizomes), relatively slender and drooping culm apices, and branching habits. Consequently, they established the separate genus *Neosinocalamus* Keng f. to emphasize its morphological distinctiveness [34]. Conversely, another school of thought maintained that *B. emeiensis’*s caespitose growth habit, inflorescence, and fundamental spikelet structure aligned more closely with *Bambusa*. They contended that *B. emeiensis* should be treated as a distinctive member of *Bambusa*, with its unique slender culm morphology representing an evolutionary adaptation to specific habitats rather than a characteristic sufficiently distinct to warrant generic separation [35,36]. Subsequent molecular phylogenetic studies consistently revealed that molecular markers from *B. emeiensis* were nested within the core clade of *Bambusa*, providing robust evidence for its inclusion in that genus [37].

Morphological observations of floral organs in this study clearly demonstrated that *B. emeiensis* possessed the typical floral character combination of *Bambusa*: six stamens, three prominent obovate lodicules, and a pistil with a short style and long stigmas. These characteristics stood in stark contrast to the vegetative traits emphasized by the proposal for its recognition as a separate genus. LDA results further confirmed that the floral morphology data of *B. emeiensis* fell entirely within the discriminant space of *Bambusa*, with high classification confidence. Therefore, the detailed morphological description of *B. emeiensis* flowers in this study supported its inclusion within *Bambusa*. This finding was highly congruent with molecular phylogenetic evidence [18], collectively confirming the systematic position of *B. emeiensis* within *Bambusa*. This study also demonstrated the utility of PCA and LDA in bamboo taxonomy.

Bamboo has a long flowering cycle and rarely blooms. Many bamboo species rely solely on vegetative characteristics for identification at the time of publication, lacking descriptions of floral organs. This paper described the floral organ characteristics of 23 bamboo species, among which five species—*Pleioblastus fortunei*, *Gigantochloa* sp., *Gigantochloa* sp., *Phyllostachys sulphurea*, and *Fargesia yuanjiangensis*—exhibited floral morphological features not previously recorded in *Flora of China*, *Illustrated Flora of Bambusoideae in China*, or other literature. Morphological descriptions of these bamboo floral organs provided critical evidence for future classification and identification. Additionally, these new descriptions offered essential morphological evidence for revising bamboo taxonomic systems. For example, the stamen number in *Gigantochloa* (three to six) formed a distinct contrast with that of *Bambusa* and other genera (typically six), potentially suggesting divergence in their evolutionary pathways [38].

In the BDG complex group, where controversy over genetic introgression existed in molecular systematics, floral traits demonstrated stronger taxonomic stability. Species of *Bambusa* consistently possess six stamens and pistils with short styles/long stigmas, while *Dendrocalamus* exhibits pistils with long styles/long stigmas and completely lacks lodicules. The uniqueness of *Gigantochloa* was reflected in the plasticity of stamen number (three to six) and ovate-lanceolate lodicules, even among taxa exhibiting molecular marker overlap [15]. These morphological characteristics reliably distinguished *Gigantochloa* from *Bambusa* and *Dendrocalamus*.

In this study, stamen number was found to be a stable diagnostic trait at the generic level. All *Bambusa*, *Dendrocalamus*, and *Schizostachyum* species consistently exhibited six stamens, whereas *Phyllostachys*, *Pleioblastus*, and *Fargesia* species possessed three (Table 2). This pattern aligns with previous reports for the genera examined [15,39,40], reinforcing the reliability of stamen count as a morphological marker in bamboo taxonomy. Notably, *Gigantochloa* showed intrageneric variation (three or six stamens). The underlying mechanism for this evolutionary reduction in number remains unreported and requires further investigation.

In summary, this study, through a comparative morphological investigation of bamboo floral organs in the southwestern region, not only revealed the diversity and taxonomic significance of floral structures across different bamboo species but also provided novel morphological evidence for establishing intergeneric classification systems. Future research could expand the sample size to include more bamboo species and broader geographical distributions, thereby offering a more comprehensive understanding of floral diversity and taxonomic implications.

## 4. Materials and Methods

### 4.1. Plant Materials

The bamboo flower materials analyzed in this study were all obtained through field collection. They were primarily gathered from the Yunnan and Guizhou provinces in Southwest China from 2010 to 2024, encompassing 23 distinct bamboo species (seven genera) with their floral organ specimens (Table 6).

Two *Gigantochloa* specimens whose floral morphology has never previously been documented were included and are temporarily designated as *Gigantochloa* sp. 1 and *Gigantochloa* sp. 2. Voucher specimens have been deposited in the Herbarium of Southwest Forestry University (SWFC), Kunming, China, under voucher numbers SWFC0072425 and SWFC0072426, respectively. Each voucher contains detailed locality, collection date, collector information, and high-resolution photographs and is publicly available for examination.

### 4.2. Methods

Freshly bloomed mature florets were selected and collected. The spikelets and floral branches were excised and immediately immersed in FAA fixative solution (50% ethanol: formaldehyde: glacial acetic acid = 18:1:1) and then transported to the laboratory for vacuum degassing and preservation. The fixed spikelets and florets were dissected under a stereomicroscope (Olympus HO11, Evident Scientific Tokyo, Tokyo, Japan).

Florets were sequentially detached from the spikelets, and each floral organ was observed under an optical microscope. Photographic documentation and morphometric analysis of individual floral structures were performed using the 2D measurement software DS-3000. Morphological data from all organs were analyzed in IBM SPSS Statistics 21 to calculate means, standard deviations, and Z-score normalization, with the 20 flowers used for each indicator sampled from three clumps (nine individual bamboos in total). Subsequently, principal component analysis (PCA) was performed using Origin 2024, followed by linear discriminant analysis (LDA) using IBM SPSS Statistics 21. Descriptive traits (e.g., spikelet shape, ovary shape) were quantified through coding, while numerical traits (e.g., spikelet dimensions, lemma/palea lengths) retained original measurements. A total of 20 spikelets from each bamboo species were selected for measurements of morphological indicators.

## 5. Conclusions

This study systematically investigated the morphological diversity and taxonomic significance of floral organs in 23 bamboo species from Southwest China through systematic morphological observation and multivariate statistical analyses, including principal component analysis and linear discriminant analysis. The results confirmed the importance of floral characteristics as stable taxonomic markers. Core traits screened by PCA—spikelet morphology, palea length, and stamen number—collectively accounted for 84.6% of the observed variance. LDA further validated the reliability of these traits, achieving an overall genus-level classification accuracy of 95.7%. This demonstrated the value of multivariate statistical methods like PCA and LDA for bamboo classification and provided objective, quantifiable morphological criteria for genus delimitation, greatly enhancing the practicality of field identification.

## Figures and Tables

**Table 1:** Characteristics of florets across diverse bamboo species.

Genus	Species	Spikelet Shape	Spikelet Length (cm)	Spikelet Width (mm)	Rachilla Type	Rachilla Length (mm)	Lemma Shape	Pubescence on Lemma Margins	Lemma Length (mm)	Pubescence on Palea Margins	Palea Length (mm)	Pistil Type	Ovary Shape	Stigmas Number	Stamen Number	Lodicule Number	Lodicule Shape
*Bambusa*	*Bambusa sinospinosa*	Linear lanceolate	2.7–3.8	2.7–4.1	Nearly hollow	3.8–4.8	Ovate-lanceolate	No	7.3–8.2	Yes	7.1–9.0	Long style and short stigma	Obovate	3	6	3	Obovate
	*Bambusa ventricosa*	Linear lanceolate	4.9–5.9	3.0–4.5	Nearly hollow	1.9–3.1	Ovate-lanceolate	No	9.2–10.5	Yes	9.7–11.3	Short style and long stigma	Obovate	3	6	3	Obovate
	*Bambusa eutuldoides* var. *vviridivittata*	Linear lanceolate	2.8–3.8	4.7–7.5	Nearly hollow	2.7–3.7	Ovate-lanceolate	No	9.4–11.0	Yes	9.7–11.1	Short style and long stigma	Obovate	2	6	3	Irregular
	*Bambusa tuldoides*	Linear lanceolate	3.4–4.6	2.4–4.0	Near-solid	2.8–4.2	Ovate-lanceolate	No	11.5–13.5	Yes	10.7–12.9	Short style and long stigma	Obovate	3	6	3	Obovate
	*Bambusa textilis*	Linear lanceolate	2.9–4.1	2.9–5.3	Nearly hollow	3.2–4.8	Ovate-lanceolate	No	12.3–14.5	Yes	12.3–13.7	Short style and long stigma	Obovate	3	6	3	Spoon-shaped, obovate
	*Bambusa rigida*	Linear lanceolate	2.1–4.3	4.8–7.2	Nearly hollow	2.7–3.7	Ovate-lanceolate	No	11.7–13.3	Yes	9.7–10.9	Long style and short stigma	Obovate	3	6	3	Semi-spoon-shaped, Oblanceolate
	*Bambusa rutila*	Linear lanceolate	6.2–7.7	2.4–3.9	Nearly hollow	2.3–3.1	Ovate-lanceolate	No	9.2–11.1	Yes	11.0–12.6	Short style and long stigma	Obovate	3	6	3	Ovate
	*Bambusa cerosissima*	Long-ovate	1.8–3.2	6.6–8.0	Nearly hollow	3.5–5.0	Broadly ovate	No	9.3–9.9	No	9.0–10.2	Short style and short stigma	Obovate	3	6	3	Ovate
	*Bambusa intermedia*	Linear lanceolate	4.3–5.1	2.0–3.2	Nearly hollow	1.8–2.8	Ovate-lanceolate	No	11–12.2	Yes	12.2–14.4	Short style and short stigma	Obovate	1	6	3	Elongated-lanceolate
	*Bambusa emeiensis*	Long oval shape	1.9–3.5	5.1–8.1	Near-solid	1.5–2.9	Broadly ovate	Yes	8.2–9.8	Yes	7.8–9.4	Long style and long stigma	Obovate	3	6	3	Oblong-lanceolate
*Gigantochloa*	*Gigantochloa* sp.	Lanceolate	2.5–2.9	1.4–2.9	Near-solid	3.1–4.3	Oblong-lanceolate	No	6.7–8.5	Yes	5.2–6.2	Short style and long stigma	Obovate	3	3	3	Ovo-lanceolate
*Gigantochloa*	*Gigantochloa*	Oblong	1.9–2.6	5.4–6.8	Near-solid	4.56 ± 0.541	Broadly ovate	Yes	8.4–10.3	Yes	7.0–8.6	Short style and long stigma	Obovate	3	6	3	Ovo-lanceolate
*Pleioblastus*	*Pleioblastus fortunei*	Linear lanceolate	6.7–7.1	1.4–3.5	Near-solid	7.11 ± 0.352	Lanceolate	No	12.8–14.4	Yes	11.9–13.3	Short style and long stigma	Elliptical	3	3	3	Oblong-lanceolate
*Dendrocalamus*	*Dendrocalamus sinicus*	Ovate	3.0–3.5	6.2–8.1	Near-solid	2.67 ± 0.086	Broadly ovate	Yes	24.3–25.7	Yes	24.7–26.3	Long style and long stigma	Globular	1	6	0	/
	*Dendrocalamus giganteus*	Ovate	2.5–2.8	4.5–6.1	Near-solid	0.59 ± 0.017	Broadly ovate	Yes	9.3–11.1	Yes	9.1–10.9	Long style and long stigma	Globular	1	6	0	/
	*Dendrocalamus fugongensis*	Ovate	2.5–3.2	5.7–8.1	Near-solid	1.03 ± 0.210	Broadly ovate	Yes	11.5–12.9	Yes	11.5–12.9	Long style and long stigma	Globular	1	6	0	/
	*Dendrocalamus hamiltonii*	Ovate	0.5–1.2	4.9–7.0	Near-solid	1.2 ± 0.073	Broadly ovate	Yes	7.7–10.3	Yes	8.4–10.0	Long style and long stigma	Globular	1	6	0	/
*Phyllostachys*	*Phyllostachys sulphurea*	Narrowly lanceolate	2.3–3.2	0.6–2.5	Near-solid	5.31 ± 0.351	Lanceolate	Yes	18.6–20.0	Yes	19.7–21.3	Long style and short stigma	Obovate	1	3	3	Lanceolate
	*Phyllostachys glauca*	Narrowly lanceolate	3.1–3.6	1.5–3.1	Near-solid	2.53 ± 0.0231	Lanceolate	Yes	19.1–21.3	Yes	15.5–17.5	Long style and short stigma	Obovate	1	3	3	Lanceolate
	*Phyllostachys nigra*	Lanceolate	2.2–3.4	3.8–5.2	Near-solid	3.29 ± 0.294	Lanceolate	Yes	15.4–16.6	Yes	14.1–15.3	Long style and short stigma	Obovate	1	3	3	Obovate
*Schizostachyum*	*Schizostachyum brachycladum*	Lanceolate	2.3–3.1	2.5–4.0	Near-solid	2.25 ± 0.0621	Ovoid-caudal pointed	No	10.2–12.4	No	6.6–8.6	Long style and short stigma	Broadly ovate	1	6	3	Oblong-lanceolate
*Fargesia*	*Fargesia yuanjiangensis*	Linear lanceolate	3.1–4.2	1.8–3.1	Near-solid	5.15 ± 0.754	Lanceolate	Yes	8.8–10.0	Yes	7.8–9.6	Short style and long stigma	Obovate	2	3	3	Ovate
	*Fargesia fungosa*	Lanceolate	3.4–4.1	1.9–3.3	Near-solid	4.46 ± 0.544	Lanceolate	Yes	16.4–17.8	Yes	11.1–13.3	Short style and long stigma	Elliptical	2	3	3	Lanceolate

**Notes:** All quantitative traits are presented directly as the 95% measurement interval, obtained by ranking the 20 spikelet observations and taking the 1st–19th values, thereby reflecting the range most relevant for taxonomy and identification.

**Table 2:** Eigenvalues, contribution rates, and cumulative contribution rates of each principal.

Principal Component	PC1	PC2	PC3	PC4	PC5
Load factor					
Spikelet morphology	0.3564	−0.1738	0.1103	−0.012	0.1417
Spikelet length	−0.2452	−0.0605	−0.3522	−0.1068	−0.5006
Spikelet width	0.1478	0.3505	−0.0126	0.0412	0.4417
Lemma length	0.1787	−0.2805	−0.4223	−0.2799	0.2219
Lemma shape	0.347	−0.1147	0.1759	−0.0825	−0.2498
Pubescence on lemma margins	0.3177	−0.1593	0.0118	0.3703	0.2023
Palea length	0.1643	−0.2022	−0.5546	−0.2395	0.1897
Pubescence on palea margins	0.0047	−0.084	−0.4163	0.6505	−0.1439
Ovary shape	0.3048	0.1116	0.12	−0.3274	−0.4039
Stigma number	−0.3313	0.1095	0.0532	0.2175	0.0282
Pistil type	0.2315	0.2922	−0.0917	0.2311	−0.0764
Stamen number	−0.0042	0.4673	−0.0841	−0.1531	0.1395
Rachis type	0.2758	−0.242	0.2175	0.2157	−0.2017
Rachis length	−0.218	−0.295	0.0131	0.0033	−0.0512
Lodicule number	−0.3512	−0.2094	0.1482	−0.0752	0.2034
Lodicule shape	−0.0871	−0.4006	0.2668	−0.0211	0.2261
Eigenvalue	5.66771	3.7606	1.79297	1.29936	1.02581
Contribution Rate %	35.4%	23.50%	11.2%	8.1%	6.4%
Cumulative Contribution Rate %	35.4%	58.9%	70.1%	78.2%	84.6%

**Note:** The cumulative contribution rate represented the representativeness of the extracted factors for all variables. Generally, 80% was taken as the critical value. The larger the value, the stronger the representativeness. The significance of each principal component was determined by the absolute value of the loading coefficient. When studying the potential significance of the floral organs of new bamboo species in taxonomy, the absolute value of 0.35 was used as the threshold to preliminarily screen out more variables that were likely to be related to the principal components, providing a basis for more in-depth analysis in the future.

**Table 3:** Eigenvalues and variance contribution of discriminant functions.

Discriminant Function	Eigenvalue	Variance Contribution (%)	Cumulative Contribution (%)	Canonical Correlation
1	3.421	69.5	69.5	0.891
2	0.996	20.2	89.7	0.709
3	0.362	7.3	97.0	0.534
4	0.128	2.6	99.6	0.347
5	0.018	0.4	100.0	0.133
6	0.000	0.0	100.0	0.000

**Note**: LDA extracted six discriminant functions (equal to the number of genera minus one). The first two functions accounted for 89.7% of the total variance, indicating strong discriminative power.

**Table 4:** Coefficients of discriminant functions.

Trait	Discriminant Function 1	Discriminant Function 2
Spikelet morphology	0.502	−0.179
Spikelet length	−0.365	0.221
Spikelet width	0.394	0.087
Lemma length	−0.428	0.193
Pubescence on lemma margins	0.376	−0.152
Palea length	−0.519	0.205
Pubescence on palea margins	0.473	−0.251
Ovary shape	−0.398	0.324
Number of stamen	0.479	0.163
Number of lodicules	−0.358	−0.210
Lodicule shape	−0.412	0.285

**Note**: The coefficients reflect the relative importance of each core trait in genus discrimination. Traits with absolute coefficients > 0.45 are critical for classification.

**Table 5:** LDA classification matrix of discriminant results.

Actual Genus	Sample Size	Correctly Classified	Accuracy (%)
Bambusa	10	10	100.0
Gigantochloa	2	2	100.0
Pleioblastus	1	1	100.0
Dendrocalamus	4	3	75.0
Phyllostachys	3	3	100.0
Schizostachyum	1	1	100.0
Fargesia	2	2	100.0
Total	23	22	95.7

The overall classification accuracy of 23 bamboo species was 95.7%. All genera except *Dendrocalamus* achieved 100% accuracy.

**Table 6:** Collection of spikelets of different bamboo species.

Genus	Species	Flowering Time	Flowering Site
*Bambusa*	*Bambusa sinospinosa* McClure	2010.4	Bamboo Garden, Kunming Expo Park, Yunnan Province
	*Bambusa ventricosa* McClure	2012.4	Bamboo Garden, Kunming Expo Park, Yunnan Province
	*Bambusa eutuldoides* var. *Viridivittata* (W. T. Lin) L. C. Chia	2013.8	Mengla County, Xishuangbanna Dai Autonomous Prefecture, Yunnan Province
	*Bambusa textilis* McClure	2013.8	Mengla County, Xishuangbanna Dai Autonomous Prefecture, Yunnan Province
	*Bambusa tuldoides* Munro	2023.7	Rare Bamboo Garden at Southwest Forestry University in Kunming City, Yunnan Province
	*Bambusa rigida* Keng & P. C. Keng	2017.4	Rare Bamboo Garden at Southwest Forestry University in Kunming City, Yunnan Province
	*Bambusa rutila* McClure	2017.5	Rare Bamboo Garden at Southwest Forestry University in Kunming City, Yunnan Province
	*Bambusa emeiensis* L. C. Chia & H. L. Fung	2012.9	Rare Bamboo Garden at Southwest Forestry University in Kunming City, Yunnan Province
	*Bambusa cerosissima* McClure	2022.10	Chishui County Bamboo Sea National Forest Park, Guizhou Province
	*Bambusa intermedia* J. R. Xue & T. P. Yi	2014.5	Yunnan Kunming World Horticultural Expo Bamboo Garden
*Gigantochloa*	*Gigantochloa* sp. 1 (SWFC0072425)	2020.5	Mangshi, Dehong Prefecture, Yunnan Province
	*Gigantochloa* sp. 2 (SWFC0072426)	2020.5	Mangshi, Dehong Prefecture, Yunnan Province
*Pleioblastus*	*Pleioblastus fortunei* (Van Houtte ex Munro) Nakai	2015.7	Simao District, Pu’er City, Yunnan Province
*Dendrocalamus*	*Dendrocalamus sinicus* L. C. Chia & J. L. Sun	2014.7	Cangyuan Wa Autonomous County, Yunnan Province
	*Dendrocalamus giganteus* Wall. ex Munro	2012.10	Mojiang County, Pu’er City, Yunnan Province
	*Dendrocalamus fugongensis* J. R. Xue & D. Z. Li	2023.1	Fugong County, Nujiang Lisu Autonomous Prefecture, Yunnan Province
	*Dendrocalamus hamiltonii* Nees & Arn. ex Munro	2015.7	Mengla County, Xishuangbanna Dai Autonomous Prefecture, Yunnan Province
*Phyllostachys*	*Phyllostachys sulphurea* (Carrière) Riviere & C. Rivière	2013.5	Panlong District, Kunming City, Yunnan Province
	*Phyllostachys glauca* McClure	2018.7	Panlong District, Kunming City, Yunnan Province
	*Phyllostachys nigra* (Lodd. ex Lindl.) Munro	2021.8	Southwest Forestry University in Kunming City, Yunnan Province
*Schizostachyum*	*Schizostachyum brachycladum* (Kurz) Kurz	2017.10	Simao District, Pu’er City, Yunnan Province
*Fargesia*	*Fargesia yuanjiangensis* J. R. Xue & T. P. Yi	2018.7	Rare Bamboo Garden at Southwest Forestry University in Kunming City, Yunnan Province
	*Fargesia fungosa* T. P. Yi	2014.4	Yunnan Kunming World Horticultural Expo Bamboo Garden

## Data Availability

The datasets generated during and/or analyzed during the study are available from the corresponding author upon reasonable request.
